# Leptomeningeal Metastasis: A Rare Complication of Non-small Cell Lung Cancer

**DOI:** 10.7759/cureus.58209

**Published:** 2024-04-13

**Authors:** Reid Schalet, Lindsey Rae, Linda Lesky, William Gesztes

**Affiliations:** 1 Department of Internal Medicine, George Washington University, Washington, D.C., USA; 2 Department of Pathology, George Washington University, Washington, D.C., USA

**Keywords:** leptomeningeal metastases, systemic lupus erythematosus, metastatic non-small cell lung cancer, autoimmune disease, non-small cell lung carcinoma (nsclc), leptomeningeal carcinomatosis (lmc)

## Abstract

Leptomeningeal metastasis (LMM) is a rare complication of non-small cell lung cancer (NSCLC) that can present with a range of neurological symptoms depending on the site(s) of metastatic involvement. We present a case of a 54-year-old woman who was initially diagnosed with suspected inflammatory neuritis secondary to a known systemic lupus erythematosus (SLE) diagnosis after presenting with multiple months of progressive neuro-ophthalmologic symptoms; however, she was eventually diagnosed with LMM secondary to a previously undiagnosed NSCLC. This case both underscores the challenges of diagnosing LMM due to its nonspecific presentation, as well as highlights the importance of including LMM in the differential diagnosis for patients presenting with vague neurological symptoms in the context of another inflammatory disease process.

## Introduction

Leptomeningeal metastasis (LMM) occurs when malignant cells infiltrate the membranes surrounding the brain and spinal cord, most commonly due to the hematogenous spread of acute leukemias, breast and lung carcinomas, or melanoma [1]. As the cancer cells spread to the leptomeninges, normal central nervous system (CNS) functioning is disrupted, leading to an array of neurological symptoms such as headaches, changes in mental status, and visual disturbances [1]. Differentiating LMM from other neurological conditions, specifically neurological manifestations of systemic lupus erythematosus (SLE), can be extraordinarily difficult due to their nonspecific and overlapping clinical presentations, leading to delayed diagnosis and treatment. Here, we report a case of a 54-year-old woman with a history of SLE who presented in the setting of diplopia and cranial nerve enhancement on MRI who was initially treated for lupus neuritis but was ultimately found to have LMM from an undiagnosed non-small cell lung cancer (NSCLC). This case highlights the challenges of diagnosing LMM in the setting of vague neuro-ophthalmological complaints within the context of a known autoimmune condition.

## Case presentation

A 54-year-old woman with a prior history of tobacco use and known SLE that was well-controlled with weekly injections of Belimumab presented with a two-month history of worsening neuro-ophthalmological symptoms. She experienced her first episode of diplopia, slurred speech, headache, and mild confusion in March, where an MRI head was ordered by her ophthalmologist that showed positive enhancement of cranial nerves (CNs) IV and V. She was started on a tapering dose of prednisone for two weeks. After minimal improvement, she presented to the emergency department (ED) with complaints of worsening diplopia and left trigeminal neuropathy. An MRI of the face, neck, and orbit was performed, which showed a normal appearance of the optic nerves and optic chiasm with nonspecific, mild chronic changes in the cerebral hemispheres. The patient was started on intravenous (IV) solumedrol with reported improvement in symptoms. Given this subjective improvement, she was discharged three days later with a presumed diagnosis of inflammatory neuritis secondary to SLE.

**Figure 1:**
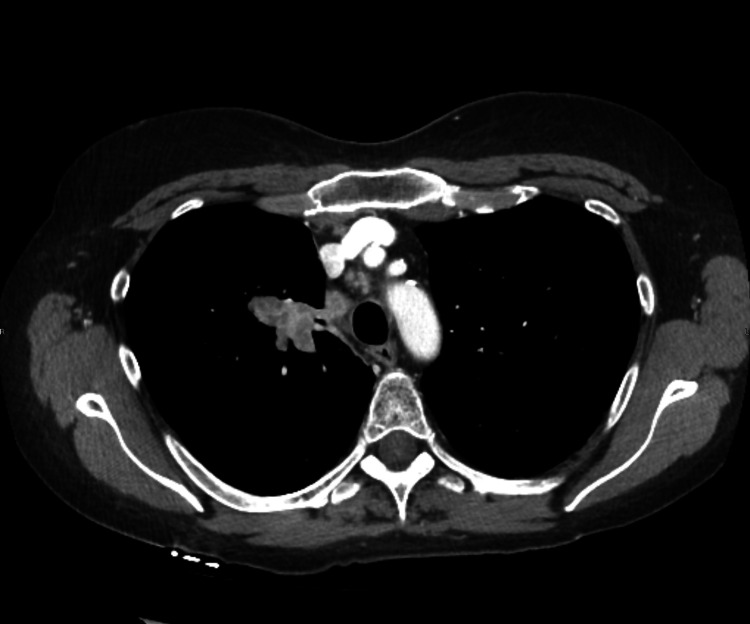
Contrast CT scan of the chest. Contrast CT of the chest revealed a 5.8 cm x 3.8 cm x 2 cm spiculated right upper lobe mass. The mass abuts the right upper lobe bronchus with narrowing of a posterior segmental bronchus.

**Figure 2:**
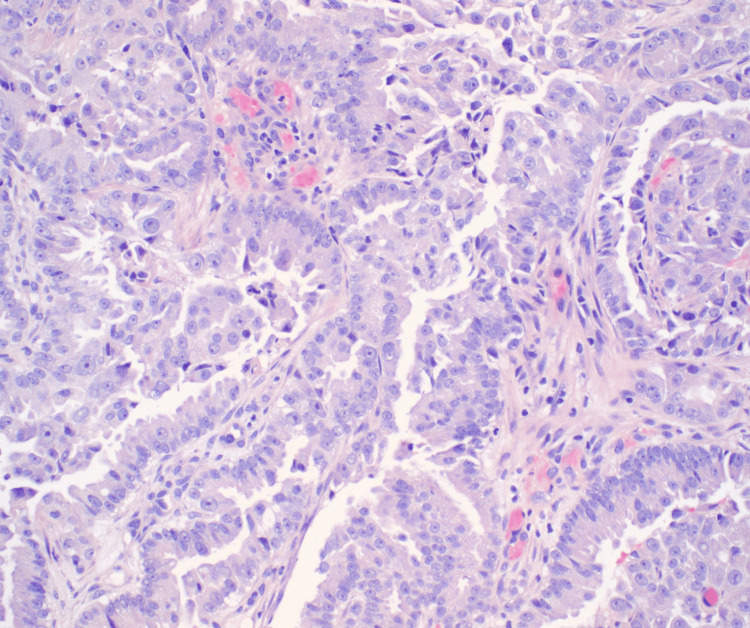
Lung biopsy, hematoxylin and eosin (H&E), x200. Transbronchial biopsy of the right upper lobe lesion with morphologic features consistent with well-to-moderately differentiated adenocarcinoma on H&E showing acinar/papillary growth pattern and nuclear atypia.

**Figure 3:**
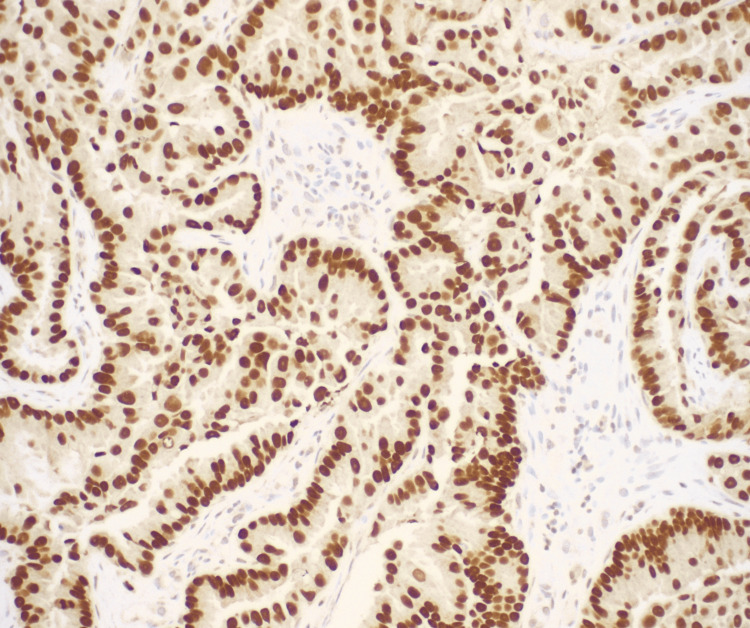
Lung biopsy, TTF-1, x200. Thyroid transcription factor 1 (TTF-1; also known as NKX2-1) immunohistochemistry (IHC) shows diffuse and marked nuclear positivity supporting a diagnosis of lung primary. p63 and p40 IHC stains were negative, precluding squamous cell carcinoma.

**Figure 4:**
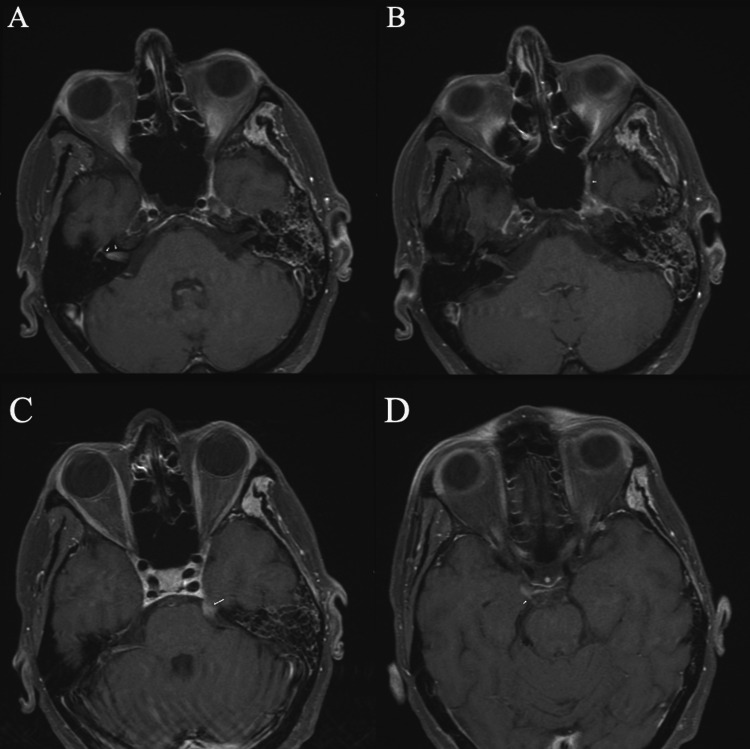
MRI of the brain with contrast revealing enhancement in multiple cranial nerves. (A) Linear post-contrast enhancement (*see arrows*) involving the right seventh and eighth nerve complex within the internal auditory canal, the basal turn of the right cochlea, and discontinuous enhancement of the right seventh cranial nerve up to its mastoid segment. (B) Linear post-contrast enhancement (*see arrow*) involving the V3 segment of the left trigeminal nerve. (C) Nodular enlargement with enhancement (*see arrow*) of the left trigeminal nerve in its cisternal segment. (D) Linear post-contrast enhancement (*see arrow*) involving the right third cranial nerve.

**Figure 5:**
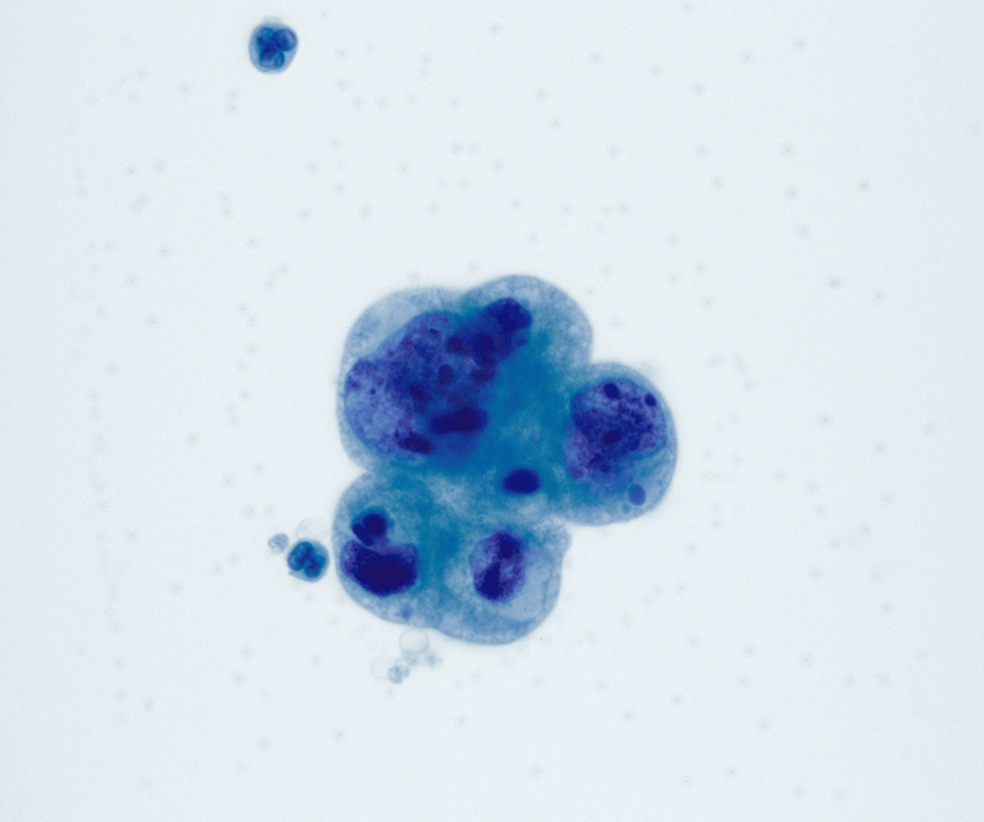
CSF cytology, Thinprep, x500. Cerebrospinal fluid (CSF) obtained via lumbar puncture (Thinprep) revealed markedly enlarged atypical cells aggregating in a small, tight cluster with finely vacuolated cytoplasm. Some cells displayed an increased nucleus-to-cytoplasmic ratio, along with round-to-irregular nuclear contours and coarse chromatin clumping or nuclear hyperchromasia. This was observed in a background of polymorphonuclear neutrophils (PMNs).

The patient then returned to the ED a month later with worsening diplopia, left facial numbness, slurred speech, and an unsteady gait. In the ED, a CT angiography (CTA) of the head and neck was ordered, which showed a 3.8 cm medial right upper lobe pulmonary consolidation, multiple pulmonary nodules, and mediastinal and supraclavicular lymphadenopathy. A CT of the chest, abdomen, and pelvis was performed, which showed a 5.8 cm spiculated right upper lobe lung mass, with numerous pulmonary nodules, bilateral supraclavicular lymphadenopathy, and multiple osseous lesions - consistent with metastasis from primary lung cancer (Figure 1). Endobronchial ultrasound with biopsy officially confirmed the diagnosis of epidermal growth factor receptor (EGFR)-wild-type lung adenocarcinoma, with suspected leptomeningeal metastasis (Figures 2-3). This was further illustrated by the enhancement of the right CNIII, left CNV, right CNVII, right CNVIII, and bilateral optic nerves seen on a repeat MRI of the brain and orbit (Figure 4). An outpatient cerebral spinal fluid (CSF) cytology study later revealed scattered atypical cells consistent with metastatic disease (Figure 5). The patient was recommended to continue daily oral prednisone treatment and was referred to oncology for further evaluation and treatment. In addition, the patient was recommended to follow up with her rheumatology team. A decision was made along with her oncologist to initiate immediate chemotherapy treatment with six cycles of Carboplatin and Pemetrexed.

## Discussion

LMM is a rare complication of lung carcinomas, characterized by a variety of clinical symptoms dependent on the site and extent of metastatic disease [1]. Within the United States, 57% of cases of NSCLC progress to metastatic disease, with the CNS remaining one of the most common sites of metastases [2,3]. Among these patients with NSCLC complicated by CNS involvement, approximately 4%-7% of patients have LMM [4]. The age of patients with a solid malignancy complicated by LMM remains consistent, with a majority of patients diagnosed in their late fifties [5]. In the case of our patient, the presenting vague neuro-ophthalmological symptoms with MRI findings of cranial nerve enhancement resulted in a presumptive diagnosis of lupus neuritis. However, the progression of worsening symptoms despite steroid treatment suggested an alternative diagnosis.

The case underscores the challenges associated with diagnosing LMM, particularly in patients with existing autoimmune or chronic inflammatory conditions due to overlapping clinical presentations. This case also serves as a reminder of the significance of considering LMM as a potential diagnosis in the setting of nonspecific neurological symptoms, including in individuals with an underlying autoimmune disease. The importance of this consideration stems from the poor prognosis associated with LMM and the need for early intervention. LMM in the setting of NSCLC is associated with an expected survival of three to 11 months after initial diagnosis and treatment [6]. Therefore, clinicians must remain aware of possible malignancies, even when symptoms might seem explainable by existing autoimmune diseases. Prompt evaluation of LMM may avoid delays in diagnosis and potentially improve patient outcomes.

## Conclusions

LMM is a rare but devastating sequela of lung carcinomas that can present with an array of symptoms depending on the site and extent of metastatic involvement. This case highlights the challenges associated with diagnosing LMM, especially in the context of patients with preexisting autoimmune or chronic inflammatory conditions due to overlapping clinical manifestations. In these patients, such as the woman presented in this case, LMM must be considered in the context of nonspecific neurological symptoms, given its poor prognosis and need for early intervention. Clinicians should consider malignancy as a possible differential diagnosis even if the symptoms can be attributed to complications of an already established autoimmune disease to prevent a delay in diagnosis.
